# Open field test for the assessment of anxiety-like behavior in *Gnathonemus petersii* fish

**DOI:** 10.3389/fnbeh.2023.1280608

**Published:** 2024-01-10

**Authors:** Petra Horka, Veronika Langova, Jan Hubeny, Karel Vales, Ivana Chrtkova, Jiri Horacek

**Affiliations:** ^1^Institute for Environmental Studies, Faculty of Science, Charles University, Prague, Czechia; ^2^National Institute of Mental Health, Klecany, Czechia; ^3^Third Faculty of Medicine, Charles University, Prague, Czechia; ^4^Department of Circuit Theory, Faculty of Electrical Engineering, Czech Technical University in Prague, Prague, Czechia

**Keywords:** open field, motor behavior, exploratory behavior, *Gnathonemus petersii*, EOD, anxiety, thigmotaxis

## Abstract

The open field test (OFT) is a basic and most widely used test for investigation in animal studies of the neurobiological basis of anxiety and screening for novel drug targets. Here, we present the results of an OFT for weakly electric fish *Gnathonemus petersii.* This study aimed to describe the behavioral response of *G. petersii* exposed to an OFT, simultaneously with an evaluation of electrical organ discharges (EOD), to determine whether any association between EOD and patterns of motor behavior in the OFT exists. Treatment of OFT activity and its temporal patterning was assessed for the whole 6-min trial as well as per-minute distributions of activity using a near-infrared camera and an EOD data acquisition system. Our results demonstrated that the time spent, distance moved, and time of activity were significantly higher in the periphery of the OFT arena. The zone preference pattern over the 6-min test session showed that *G. petersii* prefer the outer zone (83.61%) over the center of the arena (16.39%). The motor behavior of fish measured as distance moved, active time, and swim speed were correlated with the number of EODs; however, no relationship was found between EOD and acceleration.

## 1 Introduction

The open field test (OFT) is a basic and extensively used animal model of adaptive behavior in a new environment (Stewart A. et al., 2012; Stewart A. M. et al., 2012). It generally provides information on spontaneous locomotor activity and anxiety-related emotional behavior. Hence, it is widely used for investigation in studies of the neurobiological basis of anxiety and screening for novel drug targets and anxiolytic compounds (Walsh and Cummins, 1976; Prut and Belzung, 2003; Kraeuter et al., 2019). For example, changes in locomotory activity (hyperlocomotion) are a behavioral syndrome characterized by excessive movement and activity, which can be used as a behavioral model for studying the neurobiology of psychotic-like states, or as a model for studying the neural mechanisms underlying the positive symptoms of schizophrenia (Powell et al., 2009; Langova et al., 2023).

The OFT is suitable for assessing motor activity as a reaction to an unknown environment, i.e., locomotion motivated by exploration (Jänicke and Coper, 1996). Various mechanisms may guide exploration; however, the primary factors that motivate exploratory behavior include reactions to novelty, surprise, and curiosity (Burns, 2008; Finger et al., 2016; Franks et al., 2023). The other reason for exploration is that an open field is a stressful environment for the animal, whose escape is prevented by surrounding walls (Jänicke and Coper, 1996). While the OFT is a valuable tool for assessing spontaneous locomotor activity and anxiety-related behavior, it also has limitations compared to other behavioral tests. For example, it may not provide specific insights into the other cognitive functions such as learning and memory, or social preference, which can be obtained by other methods that use novel stimuli, anxiety-inducing stressors, or memory-modulating agents (Stewart A. et al., 2012; Stewart A. M. et al., 2012). In behavioral research, the OFT has been mostly used in rodents and fish (Schmitt and Hiemke, 1998; Donald et al., 2011; Larke et al., 2017); however, many other new model species have emerged, including pigs (Haigh et al., 2020) and humans (Kallai et al., 2007; Gromer et al., 2021). Recently, fish models have been widely used in neuroscience and pharmacological research, mainly for their diverse range of behavior, similar to what is seen in mammals, including social interactions, communication, and responses to environmental conditions (Oliveira et al., 2011; Okuyama et al., 2020). This behavior is mediated by complex neural circuits and involve the same neurotransmitters and hormones that are conserved across fish and other vertebrates, including those involved in mammalian emotional and cognitive processes (Eaton et al., 1977; Langova et al., 2020). In addition, many fish species have been shown to display analogous behavior to that seen in mammals, such as aggression or anxiety (Oliveira et al., 2011). In fish, the OFT has been presented in various species, mainly zebrafish *Danio rerio* (Maximino et al., 2010a,b; Stewart A. et al., 2012; Stewart A. M. et al., 2012; Mac Rae and Peterson, 2015; Yue and Kandel, 2020), goldfish *Carrasius auratus* (Yue and Kandel, 2020), and three-spined sticklebacks *Gasterosteus aculeatus* (Hofmann and Fernald, 2000). Although these models are suitable for various pharmacological tests, other fish models provide a unique opportunity to expand our knowledge of the neural and behavioral mechanisms underlying the development of psychotic-like symptoms.

The major pitfall of the current approaches in the animal modeling of various mental disorders is their inability to evaluate communication aberrancies. For example, modeling certain specific language-based symptoms such as verbal hallucination, disorganized speech, and delusions is completely restricted, and further novel approaches are required (Kunze and Wezstein, 1988; Langova et al., 2020). Therefore, the elephant-nose fish *G. petersii* may be included among these newly emerging species, because it possesses an electric organ in its tail emitting electric organ discharges (EOD) used for electrolocation and communication (Moller, 1995; Cain and Malwal, 2002). Weakly electric fish generate electric fields by emitting EOD from their electrocytes (Carlson, 2002). These EOD create an electric field around the fish, which is used for navigation, but also as a means of communication (Kramer and Bauer, 1976; Moller, 1995; von der Emde, 1999; Cain and Malwal, 2002; Hofmann et al., 2014). These signals differ in waveform and frequency depending on the context in which they are used, but partially they are interchangeable and can also vary between individuals (Gebhardt et al., 2012). The specific electric patterns in EOD can be influenced by various environmental factors, including social context, water quality, and perceived threats (Bell et al., 1974; von der Emde, 1993; Hanika and Kramer, 2008). These patterns may also change in reaction to stressors or unfamiliar social situations and hence can be used to evaluate anxiety-like responses. Monitoring fish EOD, locomotory activity, and their relationship provides a unique opportunity to observe and measure the impairment of cognitive functions or the induction of specific symptoms of schizophrenia. To model the altered behavior, various psychotropic drugs may be used to induce positive symptoms of schizophrenia, including, e.g., disorganized thinking and cognitive deficits, manifested via disrupted EOD signaling or delusions and hallucinations, which can be analogously manifested by hyperlocomotion and erratic movements in animals (see Langova et al., 2023).

While zebrafish and other fish animal models have well-characterized spatiotemporal behavior characteristics in novel environments, the organization of *G. petersii* OFT activity has not yet been examined in detail, although some studies do exist. For example, Hofmann et al. (2014) systematically investigated the electromotor exploratory behavior of this fish species containing a single metal object in the center of the arena. Therefore, the present study aims to evaluate the basic behavioral responses of *G. petersii* to the OFT, enriched by an evaluation of the EOD responses to fish behavior, particularly anxiety-related behavior and spatiotemporal patterns of activity, and to compare them to those found in the previously mentioned models.

The OFT is one of the most commonly used anxiety assays, exploiting the approach–avoidance conflict. The behavioral response of the animal reflects a conflict between the motivation to explore novel environments and forage for resources (approach, exploration) while evading predators and other potentially harmful threats, and the preference of the animal for protected areas (avoidance). The OFT consists of a square wall-enclosed area where the measurements involve differing types of locomotory activity (Walsh and Cummins, 1976). In zebrafish and rodent research, animals in the OFT have been found to avoid the center of the open arena and prefer places close to the walls, presumably because the latter offers protection from visually hunting predators. It may also reflect a natural tendency of most animals to avoid risk-prone situations (Treit and Fundytus, 1988; Rodgers et al., 1997; Ramos et al., 2008; Seibenhener and Wooten, 2015). Greater time spent in the outer zones of the maze is recorded as thigmotaxis and indicates anxiety-related behavior (Belzung and Griebel, 2001; Carola et al., 2002; Prut and Belzung, 2003; Richendrfer et al., 2012). Both rodents and zebrafish demonstrate habituation responses in the OFT, changing their exploration behavior as they explore the novel tanks (Bolivar et al., 2000; Champagne et al., 2010; Stewart A. et al., 2012; Stewart A. M. et al., 2012). The most common measures in both fish and rodent models are time spent in the inner/outer zone of the OFT, total distance moved, and zone preference (%time spent per zone) across time and space (Bouwknecht and Paylor, 2008).

The objective of the study is to provide a detailed description of the behavior of *G. petersii* in a novel environment (the OFT) and to define a parameter of behavior to enrich our knowledge in neuropharmacological research. Using an open-field maze, we aimed to evaluate their locomotory activity and thigmotaxis, which was measured simultaneously with the EOD responses emitted by the fish. To evaluate the OFT behavior and related EOD, we exposed *G. petersii* to an OFT arena for 6 min, evaluating the per-minute spatiotemporal distribution of activity and EOD 1-min intervals. We expected that the time spent in the periphery of the arena would be greater than the time spent in the center, as was observed in rodents and fish models. To evaluate *G. petersii* behavior, we particularly focused on the following tasks: (1) determine whether a novel environment induces anxiety-like behavior in *G. petersii*, (2) evaluate zone preference and the distance moved in particular zones of the arena, (3) determine how the activity of fish relates to the EOD measured, (4) what the temporal patterns of this behavior are, and (5) whether this behavior habituates over time.

## 2 Materials and methods

### 2.1 Animals and housing

A total of 25 *G. petersii* individuals (total weight 16.63 ± 6.88 g, standard length 94.15 ± 20.32 mm, total length 127.4 ± 25.1 mm) were supplied from a local distributor Vivarium (Mělník, Czech Republic). The fish were housed in a group of five individuals in a 250 L home tank under a 12:12 h light-to-dark cycle (light photoperiod was 5 a.m. to 5 p.m.) and were habituated in the facility for 1 month before testing. The home tanks were enriched with shelter, plants, and sediment. Tank water was tested twice a week for ammonia, nitrate, and nitrite levels, and 1/3 of the water was changed every 5 days. The fish were fed with chironomid larvae daily *ad libitum*. All the applicable international, national, and institutional guidelines for the care and use of animals were followed. The conditions were validated by the commission of the Ministry of Agriculture and the study was approved by the ethics committee of Charles University (registration number 19014/2019-MZE-18134, MSMT approval number 27367/2019–3).

### 2.2 Experimental design

Infrared light sources illuminating the fish were set up, and digital cameras equipped with new infrared filters and a video-tracking system that allowed us to quantify numerous parameters of the swim path of the experimental fish were used. A total of 25 individuals were tested as we expected variance in the natural responses of the fish. The measured behavioral parameters were the total distance moved (travelled), time spent, and distance moved in the inner (center) and outer (periphery) zones of the arena and the proportion of active and inactive time spent in the outer and inner zones of the arena. For the OFT maze, the inner and outer zones were divided, and time spent in the inner and outer zones was calculated and presented as a function of total time (6 min) in the maze, according to standardly used protocols (i.e., Wong et al., 2010; Cachat et al., 2011). An inner zone was defined within the distance range of 1.5 the length of the stretched-out pectoral fins of the individual fish (74.23 ± 13.25 mm) from the tank walls corresponding to the standard 1.5 widths of a rat’s body.

### 2.3 Behavioral testing

The fish were placed in the maze for tracking analysis. At the beginning of the trial, individual fish were brought from their housing room into the testing room. We tested each subject individually in a glass square arena. Two points along the body were tracked in each fish, the point was between the pectoral fins and at the beginning of the tail. Tracking accuracy was 1.3 mm. The subjects were transported to the arena using a net. The arena was filled with standard aquarium water. The size of the arena was 60 cm x 60 cm, and the water depth was 15 cm. A total of 25 specimens of *G. petersii* were individually confined in an open-field maze during a 6-min interval. After each behavioral trial, the apparatus was thoroughly cleaned and replaced with fresh water. The free movement of the individual fish was tracked, during which time the tracking software recorded the movement of the fish. Following testing, the fish were returned to their housing tank. In their environment, *G. petersii* navigates primarily by using electrolocation. The fish emit weak electric fields and react to changes in the electric fields caused by nearby objects or their surroundings. However, *G. petersii* also use other senses like vision (von der Emde and Fetz, 2007). To reduce stress and to imitate the light conditions of fish’s natural habitat, we used a camera suitable for recording in the dark. A video was recorded with an overhead 1.3 MPx near-infrared camera (IDS Imaging Development Systems GmbH, Germany), as fish favor nocturnal activity (von der Emde et al., 2008; Onyeche et al., 2013; Kareklas et al., 2016); and *G. petersii* cannot see IR light (Ciali et al., 1997). Video recordings were analyzed by LoliTrack version 4 (Loligo Systems, Denmark). The following behavioral patterns were tracked:

1. Total locomotory activity was measured as the total time and distance moved in the center and the periphery of the arena, and total locomotory activity was measured as time actively moving (active time) and resting (inactive time).
2. Thigmotaxis as a measure of the percentage of total test time that the individual fish remained close to the outer wall of the maze as an indicative of anxiety-like behavior.
3. Spatiotemporal patterns of activity
4. Duration of erratic movements and freezing, swim speed and acceleration rate
5. Correlation between EOD and active time/distance moved in fish.

All the respective treatments of OFT activity and its temporal patterning were assessed for the whole 6-min trial as well as per-minute distributions of activity. Thigmotaxis was measured as time spent (min) and distance moved (cm) within the distance range of 1.5 lengths of stretched-out pectoral fins of individual fish from the tank walls. Locomotion was measured as the percentage of time during which the fish were active (%) and as a total moved distance (cm) according to Riehl et al. (2011). Means for all individuals ± s.d. were used for the calculation of the proportion of time in particular zones of arena and arena as a whole. Time spent - time that fish spent zones of the arena (Forward swim velocities were used to calculate the fish’s acceleration). When assessing an erratic movement, we followed a protocol of Cachat et al. (2010a,b), who used manual recording of observation. As an erratic movement, a sharp, rapid change in direction or movement, or repeated darting were registered. Increasing values being indicative of higher anxiety levels. Freezing was defined as a total absence of movement, except for the gills and eyes, for 2 s or longer (Levin et al., 2007). Before each experiment, the experimental tank was drained and refilled with fresh water under constant conditions. The water conductivity was 286 ± 44μS, temperature 23.8 ± 0.5°C, and pH 7.02 ± 0.2.

### 2.4 EOD signal acquisition

The EOD were recorded using our EOD data acquisition system. The system contains three hardware layers, which are sensor electrodes, an instrumental voltage amplifier, and data acquisition. The first hardware layer is made up of Ag sensor electrodes originally designed for human electroencephalographic recording. Four sensor electrodes were placed in the corners of the experimental tank 2 cm below the water surface, and the fifth reference electrode was placed in the middle of the experimental tank 2 cm below the water surface. Four electrodes made two bipolar channels on each side of the aquarium. The difference between those two electrodes on each side was amplified and interpreted as one channel. The second hardware layer was an analogue amplifier. The Ag electrodes were wired into the amplifier with amplification set to 100. The third hardware layer was the data acquisition system. The output of the amplifier was connected to a National Instruments DAQ system model: USB-6003 data acquisition unit. The signal from the amplifier was sampled at a rate of 50,000 samples per second with a 16-bit resolution. Data were displayed and stored on a PC via the National Instruments application for data acquisition. The acquisition system was synchronized with the recording camera using a common clock cycle, which triggered the EOD signal acquisition and digital image acquisition (infrared camera system).

### 2.5 EOD signal processing

The acquired signal from each electrode was pre-processed and processed in a MATLAB environment using the fieldtrip toolbox (Oostenveld et al., 2011). Firstly, data were filtered with a high pass FIR (finite impulse response) filter with a 200 Hz cut-off frequency. Then the data were segmented into the selected time segment. In this segment, an EOD spike detection algorithm was used to detect and calculate the sum of EOD. EOD detection was performed on two bipolar channels and only the EOD with higher amplitude was used in the analysis in each EOD detection. The spike detection algorithm was designed with the following parameters: minimum peak height = 5 millivolts, minimum peak distance = 0.02 s. These parameters were used based on prior knowledge of EOD (von der Emde and Zelick, 1995) and experimental trials and recordings.

### 2.6 Data analyses

**Figure 1:**
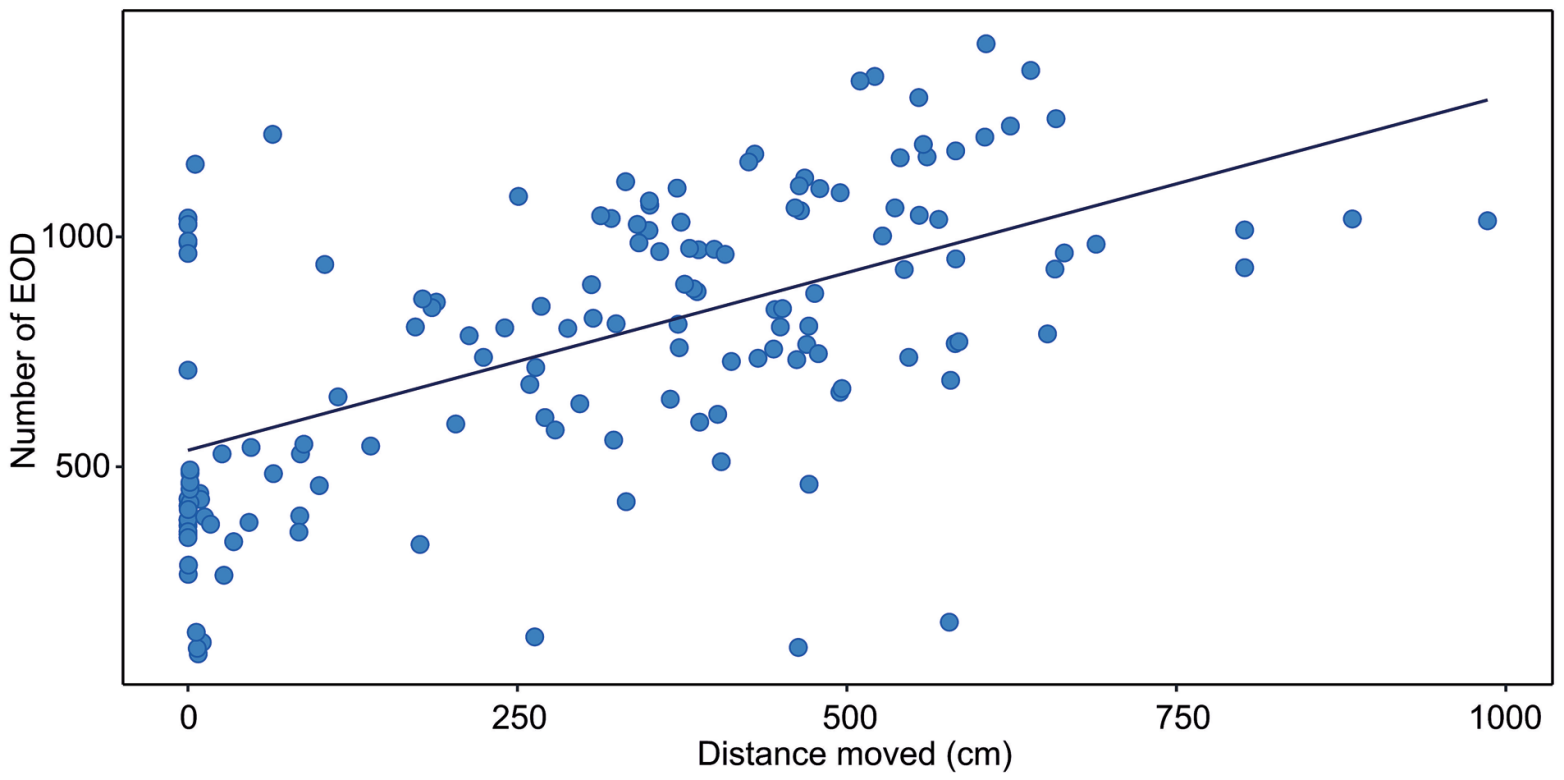
Correlation between the distance moved and the number of EOD measured in *G. petersii* OFT. The data show individuals (*n* = 25) in 6 per-minute intervals. The estimated correlation line equation: y = 53,885 + 0.077x, Kendall’s correlation coefficient *t* = 0.4; Adj. *r*^2^ = 0.328, *p* < 0.001.

**Figure 2:**
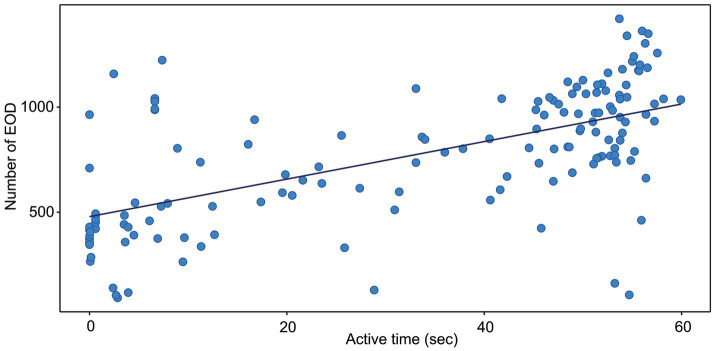
Correlation between the active time and the number of EOD measured in *G. petersii* OFT. The data show individuals (*n* = 25) in 6 per-minute intervals. The estimated correlation line equation: y = 479.0 + 8.92x, Kendall’s correlation coefficient *t* = 0.47; *z* = 8.47; Adj. *r*^2^ = 0.358, value of *p* <0.001.

*G. petersii* were subjected to the open-field maze and the total distance in centimeters of their respective tracks was combined and statistically analyzed to visualize any differences in activity. All the statistical analyses were performed using R 4.0.5 software (R Core Team, 2021). Before analysis, the data were checked for normality and homogeneity of variance. To check the test assumptions, we used the Shapiro–Wilk normality test and visual inspection of the data normality using Q-Q plots (quantile-quantile plots). The Wilcoxon rank test was used to evaluate the differences in activity patterns in the center and periphery of the arena. One-way repeated measures ANOVA and Friedman non-parametric test was used to analyze the significance of spatiotemporal patterns of behavior. Pearson’s correlation test was used to evaluate the correlation between a number of EOD and the distance moved. Kendall’s rank correlation test was used to evaluate the correlation between the number of EOD and active time as the data were not normally distributed. To generate Figures 1, 2, correlation analyses were performed between the number of EOD and distance moved and the number of EOD and active time. Six data points (one for every minute) were extracted from each fish.

The EOD frequency may be under volitional control with the sustained increase in the EOD frequency slightly preceding self-initiated movements with highly variable transition latencies (Jun et al., 2014). On the contrary, external sensory stimuli trigger an immediate movement and a near-simultaneous associated increase in the sensory sampling rate (Comas and Borde, 2010). As we were unable to detect motivation for movement activity, we did not take the possible time lag between the increase in EOD and the onset of movement into account when correlating EOD with movement activity.

## 3 Results

### 3.1 Total locomotory activity measured as total time, distance moved, and time of activity in the center and the periphery of the arena

Total locomotor activity measured as total time, distance moved, and time of activity in the center, periphery, and total area of the OFT maze was evaluated over the 6 min, and is presented in Table 1. Analysis of the proportion between distance moved and time spent in the center and the periphery of the arena revealed a greater preference for the periphery of the arena (Table 2).

**Table 1:** Time spent and swimming distance of *G. petersii* in individual zones of the OFT arena.

	Time spent in the zone (s)	Active time (s)	Inactive time (s)	Distance moved (cm)
Total area	365.5 ± 6.1	193.6 ± 123.0	172.0 ± 124.0	1807.3 ± 1246.7
Center	59.9 ± 66.1	54.1 ± 59.1	5.8 ± 8.6	578.4 ± 539.2
Periphery	305.6 ± 68.0	139.5 ± 85.6	166.2 ± 128.8	1228.9 ± 907.0

Time spent in individual zones (seconds), the ratio of active time (seconds), inactive time (seconds) and total swimming distance (cm).

**Table 2:** Proportion of time and swimming distance in individual zones of the OFT arena (mean ± s.d.); P – periphery, C – center, TA – total area of the arena.

	Time spent in the zone	Distance moved
P: TA	0.835 ± 0.18	0.734 ± 0.17
C: TA	0.165 ± 0.18	0.265 ± 0.17
C: P	0.270 ± 0.36	0.439 ± 0.37

**Figure 3:**
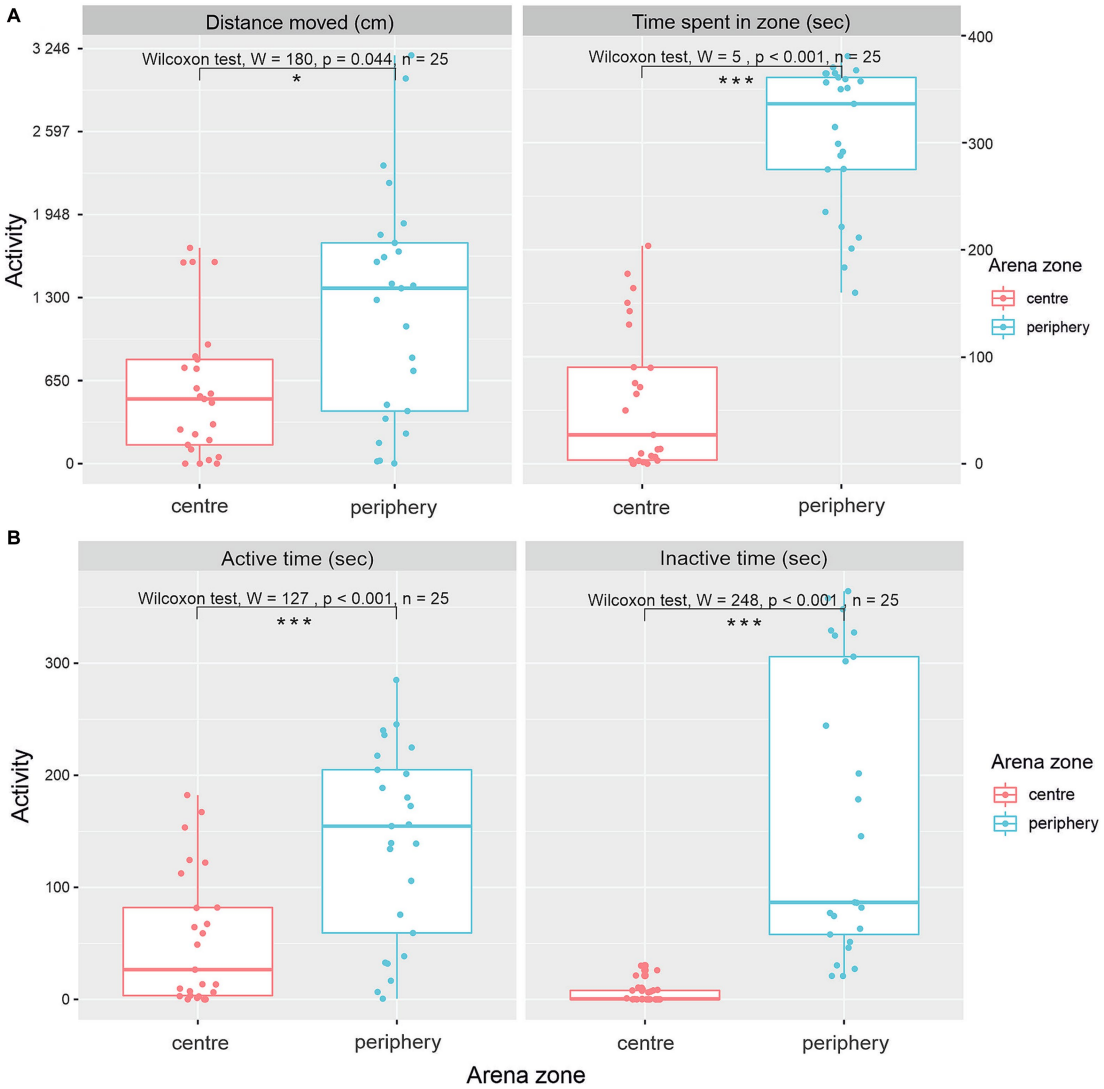
Various patterns of activity of *G. petersii* in OFT. Distance moved and total time *G. petersii* spent in the inner and outer zones of the arena **(A)**. A comparison of active and inactive time *G. petersii* spent in the center and the periphery of the arena **(B)**.

Evaluation of the distance moved in various zones of the arena (Figure 3A) showed a significantly greater distance moved in the periphery of the OFT (Wilcox-rank test, value of *p* = 0.044, W = 180, *n* = 25). The median distance moved in the center was 536.7 cm (IQR = 594.5), and the median distance moved in the periphery was 1371.6 cm (IQR = 1316.1). Differences between time spent in the center and periphery of the arena show a significantly greater preference for the periphery (Figure 3A; Wilcox-rank test value of *p* <0.001, W = 5, *n* = 25). The median time spent in the center was 27 s (IQR = 86.6), and the median time spent in the periphery was 336 s (IQR = 86.1).

Figure 3B shows patterns of activity in the center and periphery of the arena. Both active time and inactive time spent were significantly higher in the periphery of the arena in comparison to the center (Figure 3B). The median active time spent in the center was 26.5 s (IQR = 78.6), and the median active time spent in the periphery was 155.0 s (IQR = 146). The Wilcoxon rank test showed that the difference was significant (W = 127, *p* < 0.0001, *n* = 25). The median inactive time spent in the center was 0.462 (IQR = 8.0), whereas the median inactive time spent in the periphery was 86.6 (IQR = 248, W = 7, *p* < 0.0001, *n* = 25).

### 3.2 Thigmotaxis as a measure of the percentage of total test time spent in OFT zones

The percentage of time spent within each zone was further evaluated as an index of zone preference. Analysis of the zone preference pattern over the 6 min shows that *G. petersii* displays a preference for the outer zone (83.61%) over the center zone (16.39%).

### 3.3 Spatiotemporal analysis of the behavior

Temporal patterns of the behavior of *G. petersii* were assessed as a per-minute distribution of activity within each one of the OFT arenas and EOD emitted (Table 3).

**Table 3:** Total distance moved, distance moved in the center and the periphery of the OFT arena, active time, and EOD emitted (mean ± s.d.).

Zones	Distance moved (cm)			Active time (s)	EOD emitted
Time	Dist. moved total	Dist. moved center	Dist. moved periphery		(No. of. peaks)
Min 1	344.2 ± 181.6	108.9 ± 82.6	241.9 ± 118.7	29.1 ± 22.3	752.23 ± 342.3
Min 2	396.9 ± 224.8	130.9 ± 90.7	278.9 ± 174.7	33.1 ± 23.5	783.60 ± 337.9
Min 3	380.9 ± 224.9	118.9 ± 94.4	262.9 ± 196.0	34.3 ± 22.3	782.7 ± 306.7
Min 4	319.9 ± 202.1	119.9 ± 113.8	200.9 ± 142.2	30.7 ± 22.2	759.5 ± 313.7
Min 5	363.9 ± 206.9	111.9 ± 90.8	251.9 ± 177.6	32.8 ± 21.2	765.5 ± 298.5
Min 6	388.9 ± 161.8	123.9 ± 97.6	265.9 ± 156.6	35.8 ± 20.1	779.9 ± 274.0

**Figure 4:**
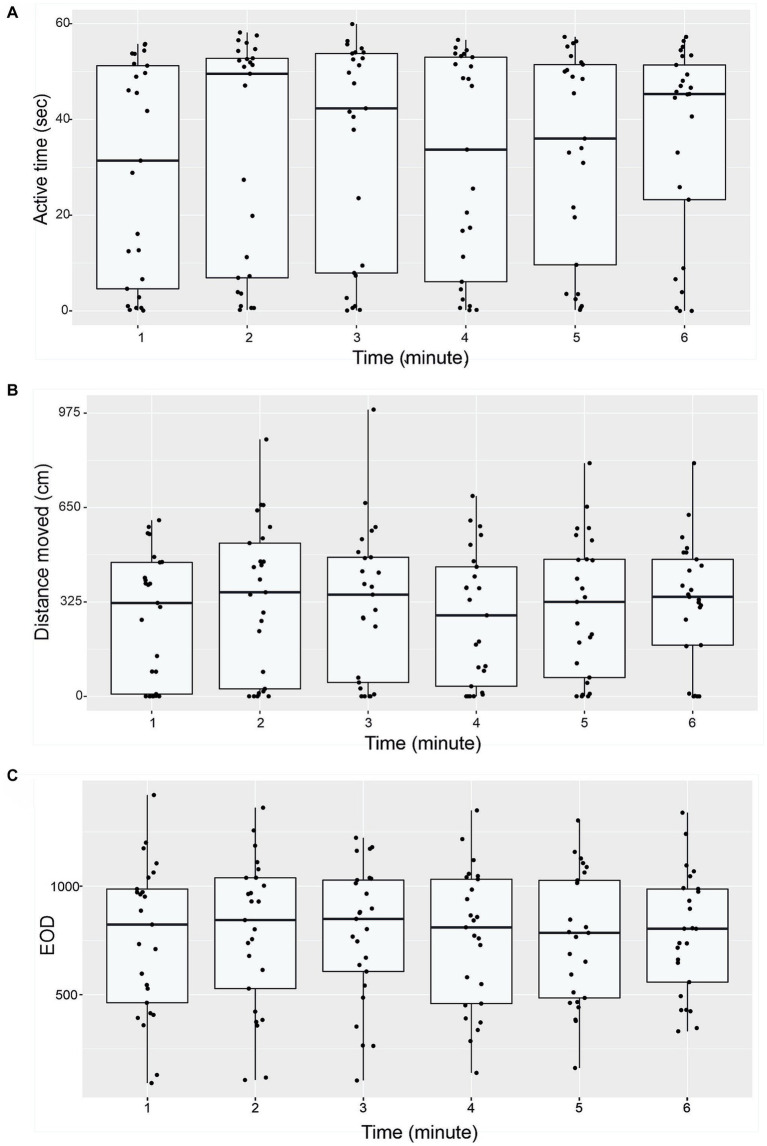
Temporal patterns of behavior of *G. petersii* (*n* = 25 individuals) in the OFT. Per-minute active time (seconds; A), distance moved (cm, B), and number of EOD measured (C). The data are presented as median, 25% and 75% percentiles for all 25 individuals.

Active time (seconds), distance moved (cm) and number of EOD in 1-min intervals are presented in Figure 4. Boxplots show median, 25 and 75% percentile and error bars for every 25 individuals in a particular time (Figure 4). No significant differences were found between different minute-to-minute intervals. Examining per-minute spatiotemporal distribution of the activity showed that the time has no effect on either active time (Friedman chi-squared = 8.06, df = 5, value of *p* = 0.153; Figure 4A), or distance moved (Friedman chi-squared = 8.57, df = 5, value of *p* = 0.127; Figure 4B), number of EOD (*F* = 1.682, df = 5, value of *p* = 0.89; Figure 4C), erratic movements (Friedman chi-squared = 3.618, df = 5, value of *p* = 0.605), or freezing duration (Friedman chi-squared = 2.694, df = 5, value of *p* = 0.747). The proportion of erratic movements per active time was 57.2%, and the proportion of freezing duration per inactive time was 40.3%. The mean per-minute frequency of erratic movements was 1.26 ± 1.24, and the frequency of freezing was 0.51 ± 0.68. The average swim speed measured was 16.6 ± 13.4 cm. s^−1^, and average acceleration rate was 45.5 ± 14.7 cm. s^−1^. The mean and standard deviation for measured variables (distance moved, active time and number of EOD) are presented in Table 3.

The correlation between the distance moved and the number of EOD is presented in Figure 1. The number of EOD significantly increased with the distance moved, with Kendall’s correlation coefficient t = 0.4. The estimated correlation line equation: y = 535.8 + 0.773x, z = 7.23, Adj. r^2^ 0.328, value of *p* = 1.12e-14. The number of EOD significantly increased with the time of activity (Figure 2), with Kendall’s correlation coefficient t = 0.468. The estimated correlation line equation y = 479.01 + 8.92x, z = 8.47, Adj. r^2^ = 0.385, value of *p* <0.001. Also, the number of EOD significantly increased with the swim speed, with Kendall’s correlation coefficient t = −0.164. The estimated correlation line equation y = 991.3–6.621x, z = −2.36, value of *p* = 0.018.

## 4 Discussion

The relation between novelty and exploration is a key issue in neurobehavioral research, as it underlies the animal’s innate behavioral organization in new environments. In modelling anxiety, the OFT is currently the most widely used test in behavioral neuroscience for screening the efficacy of different treatments and manipulations (Stewart et al., 2010; Seibenhener and Wooten, 2015). Exploratory behavior is recognized as a well-organized activity with complex spatiotemporal patterns of behavior (Champagne et al., 2010). We have adopted a task originally designed for rodents and zebrafish for *G. petersii*, where the fish were exposed to the OFT and described both spatial and temporal patterns of movement in association with the EOD emitted.

Greater time spent in the outer zones of the maze is recorded as increased thigmotaxis and is indicative of amplified anxiety-related behavior (Seibenhener and Wooten, 2015). Similarly to other studies, our results demonstrated a significant preference for the areas near the periphery of the arena. The amount of time spent and the distance travelled near the edge of the arena were significantly higher than the time/distance travelled in the center. Such results suggest that *G. petersii* behavior presents with the typical behavior that has been reported for rodents and other fish species in the interaction with a novel environment (Simon et al., 1994; Blaser and Gerlai, 2006; Lamprea et al., 2008; Stewart A. et al., 2012; Stewart A. M. et al., 2012).

The percentage of time spent within each zone over the 6-min periods shows that *G. petersii* displays a preference for the outer zone over the center zone, similar to an index of zone preference examined in the study by Champagne et al. (2010). In their study, groups of *D. rerio* spent significantly more time in the outer zone (91.80%) relative to the inner zone (8.2%). The edge preference in zebrafish larvae was observed by Richendrfer et al. (2012), who showed that the control fish spent 81.1% of the time window on the outer zone of the open-field test arena. In the study of the homebase behavior of zebrafish (Stewart et al., 2010), the fish highly preferred zones located near the walls of the tank, spending 10–40 s in the homebase zone within each minute.

Zone preference in *G. petersii* in our study had a similar pattern, i.e., center zone 16.39%; outer zone 83.61%. In animal models of schizophrenia, a manifestation of thigmotaxis can be suppressed by the induction of schizophrenia-like behavior with ketamine, which causes hyperlocomotion and associated loss of anxiety, a typical positive symptom of schizophrenia (Lipska and Weinberger, 2000; Bubeníková-Valešová et al., 2008).

The OFT is an anxiety model in animals, based on the natural behavior in unfamiliar environments, which can have variable manifestations on the continuum from the increased anxiety manifested as avoidance behavior displayed by reduced exploration, increased freezing, and/or unorganized erratic locomotion, or increased activity linked to exploration, which reflects an animal’s tendency to explore new environments (Walsh and Cummins, 1976; Maximino et al., 2010a,b; Cachat et al., 2011). This initial phase can later be followed by a change in activity that may indicate habituation to the new environment. For example, rodents usually reduce locomotion, which is followed by a repertoire of comfort behavior as they become familiar with the novel environment, whereas zebrafish appear to do the opposite (Leussis and Bolivar., 2008; Wong et al., 2010). An increase in exploratory behavior and decrease in freezing behavior are expected to occur in zebrafish (Cachat et al., 2010a).

Although the fish in our study responded to the OFT by increasing their locomotory activity measured as distance moved and active time in the periphery of the arena, no significant changes were found in between particular 1-min intervals that would indicate a temporal change in behavior associated with increased or decreased rate of exploration. A majority of the locomotory activity in *G. petersii* was associated with erratic movements, as well as with an increased rate of thigmotaxis, a behavioral profile indicative of high stress and anxiety (Egan et al., 2009; Cachat et al., 2010b). This is contrary to the patterns observed in adult zebrafish reported by Wong et al. (2010), who stated that the activity of fish increased upon initial exposure to a novel environment because of activity linked to exploration. Similar patterns were observed in zebrafish in the study of Cachat et al. (2011), which displayed fewer freezing bouts and shorter freezing duration over the 6-min testing period.

Erratic movements in mormyrid fish can be observed in various contexts and may be associated with different behaviors (Toerring and Belbenoit, 1979). However, in several fish species, erratic movement and freezing are known to be emitted in response to predators or fear-inducing stimuli (Speedie and Gerlai, 2008). According to Cachat et al. (2010b), inhibited exploration, reduced speed, and increased frequency of escape-like erratic behaviors are usually associated with higher levels of anxiety elicited by different stressors. In *G. petersii*, high duration of erratic movements can be understood as an effort to recognize as quickly as possible ways to escape from a space that is unfamiliar to the fish, and inside which they feel fear or even panic. Also, many individuals displayed freezing behavior, a typical behavior linked to anxiety, corresponding to a typical anti-predation (avoidance) behavior, where the individual does not move to reduce the probability of being seen by a predator. On the contrary, in the study of Rosemberg et al. (2011), the zebrafish were found not to freeze at all during the novel tank test. In the recorded behavior of *G. petersii*, high variability between individuals can be noticed, which is manifested by a high dispersion of the measured values of individual types of behavior. Such a behavioral response could be explained by the influence of individual variability across a ‘shyness–boldness’ continua, and innate inter-individual differences (Brown et al., 2005; Maximino et al., 2010a,b; Gebhardt et al., 2012). The above-mentioned patterns of behavior show that *G. petersii* display anxiety-related behavior similar to rodent and zebrafish models (Lamprea et al., 2008; Amar and Ramachandran, 2023), suggesting that *G. petersii* can be considered an additional psychobiological animal model useful for neurobehavioral research.

A positive correlation was found between the number of discharges and activity level measured as active time and distance moved. Agitation, including hyperlocomotion and erratic movements, can occur as a positive symptom of schizophrenia. It may be linked to heightened emotional states, anxiety, or distress (Langova et al., 2020). Also, increased motor activity (hyperlocomotion) and erratic movements can be exhibited during hallucinations or delusions. Since the increased movement activity of fish manifests itself in increased EOD, it may be assumed that the manifestations of behavioral responses to agitation (hyperlocomotion, erratic movements) would also manifest themselves in response to electrical signals. As the locomotory activity of *G. petersii* is correlated with the number of EOD, it is possible to associate these responses with observed analogues of various symptoms of psychiatric disorders, which may overall enhance the possibility of studying their mechanisms. For example, a drug that enhances boldness in an open-field test in fish may have potential therapeutic use in various contexts, e.g., fish that exhibit more boldness or reduced anxiety-like behavior in OFT may suggest that the drug has anxiolytic properties. This can then also manifest itself in different ways in the response to electric signaling, e.g., boldness in the OFT may be associated with improved exploratory behavior, which can also be associated with a different manifestation of EOD.

To examine patterns of *G. petersii* behavior, we used an overhead view. While this provides an accurate detection of behavior, further studies may utilize multiple cameras to also capture vertical exploratory activity. Also, the EOD frequency is known to increase near electrically detectable objects (Hofmann et al., 2014); however, we were not able to distinguish between EOD frequency measured near the wall-bounded periphery and the center of the arena in the current settings. Advanced settings adjustments may help clarify the relationship between locomotor activity and EOD signal near the edge and the center of the arena in future studies. Various external factors may influence the results of the behavioral manifestation of exploratory behavior in the OFT, including differences in the size of testing apparatuses, and procedural environmental factors such as temperature, conductivity, and light intensity for the tracking behavior. Individual behavior also plays a role in data variability (Riehl et al., 2011; Amar and Ramachandran, 2023). It is therefore highly recommendable to evaluate variability in the behavior of individuals in further studies, especially how the individual EOD emitted response to movement behavior, and if this behavior would display a consistent response in various other types of experimental trials.

Overall, our results suggest that the exploratory activity and level of anxiety of *G. petersii* reflect strategies observed in other fish and rodents (e.g., Lamprea et al., 2008; Wong et al., 2010; Stewart A. et al., 2012; Stewart A. M. et al., 2012), which predicts that the OFT may be a valid test of anxiety for *G. petersii*. Moreover, the study of electrical discharges provides a unique opportunity to investigate the neurobiological basis of complex behavior and neuropsychiatric disorders and can enlighten preclinical research aimed at developing new treatments for these conditions. Understanding how drugs impact the behavior and the electrical activity of the fish’s nervous system may shed light on similar mechanisms in humans. This knowledge could aid in the development of more targeted and effective treatments for anxiety and related disorders. Further research may therefore focus on investigating the specific effect of using specific anxiolytic substances on observed behavior and to test if this behavior also manifests itself in a different response of electric discharges emitted to provide more complete and reliable tests of modulation of *G. petersii* behavior.

## Data availability statement

The raw data supporting the conclusions of this article will be made available by the authors, without undue reservation.

## Ethics statement

The animal study was approved by Ethics committee of Charles University (registration number 19014/2019-MZE-18134, MSMT approval number 27367/2019-3). The study was conducted in accordance with the local legislation and institutional requirements.

## Author contributions

PH: Conceptualization, Data curation, Formal analysis, Funding acquisition, Investigation, Methodology, Project administration, Resources, Visualization, Writing – original draft, Writing – review & editing. VL: Conceptualization, Data curation, Funding acquisition, Investigation, Methodology, Writing – review & editing. JaH: Data curation, Formal analysis, Investigation, Methodology, Software, Writing – review & editing. KV: Conceptualization, Methodology, Validation, Writing – review & editing. IC: Formal analysis, Software, Writing – review & editing. JiH: Conceptualization, Funding acquisition, Investigation, Methodology, Project administration, Resources, Supervision, Validation, Writing – review & editing.
